# The Role of 18-Fluoro-2-Deoxy-Glucose Positron Emission Tomography/Computed Tomography as Response and Prognosis Predictive Factor of Concurrent Chemoradiotherapy after Induction Chemotherapy in Head and Neck Squamous Cell Carcinoma: A Prospective Study

**DOI:** 10.3390/medicina54020031

**Published:** 2018-05-15

**Authors:** Severina Šedienė, Ilona Kulakienė, Viktoras Rudžianskas, Rita Ambrazienė

**Affiliations:** 1 Medical Academy, Lithuanian University of Health Sciences, Kaunas 44307, Lithuania; kulakiene@dr.com; 2 Oncology Institute, Lithuanian University of Health Sciences, Kaunas 44307, Lithuania; viktoras.rudzianskas@lsmuni.lt (V.R.); rita.ambraziene@lsmuni.lt (R.A.)

**Keywords:** head and neck cancer, induction chemotherapy, SUVmax, hypermetabolic tumor volume

## Abstract

*Background and objectives:* The importance of induction chemotherapy (ICT) followed by concurrent chemoradiotherapy (CCRT) has been re-established in recent years aiming at fewer metastatic sites and better control of the disease. We prospectively studied the possibility of early prediction of overall survival (OS) and progression-free survival (PFS) after 3 cycles of chemotherapy with doxetacel, cisplatin and 5-fluorouracil using 18-fluoro-2-deoxy-glucose positron emission tomography computed tomography (^18^F-FDG PET/CT) in patients with head and neck squamous cell cancer. To our knowledge, this is the first such study. *Materials and Methods:* Thirty-five patients were studied. They underwent an ^18^F-FDG PET/CT examination twice: a day before ICT and 10–14 days after the last cycle of ICT. Tumor-standardized uptake value (SUVmax) and hypermetabolic tumor volume were measured on both scans. The mean age of patients was 56.5 years. Complete responses to CCRT PFS and OS were calculated. *Results*: Our results showed that a decrease of ≥30% in the SUVmax value after ICT was a prognostic factor of tumor response to PFS and OS (*p* = 0.026 and *p* = 0.021). The groups of patients with a SUVmax between 10 and 14.5 in the primary tumor on a pre-ICT ^18^F-FDG PET/CT scan had statistically shorter PFS and OS (*p* = 0.001, *p* = 0.006) when compared with other groups of patients with SUVmax less than 10 or SUVmax more than 14.5. A decrease of less than 55% of hypermetabolic tumor volume of the primary tumor was significantly related to poor prognosis in PFS and OS (*p* = 0.033, *p* = 0.017). *Conclusions:* SUVmax and hypermetabolic tumor volume measured on ^18^F-FDG PET/CT after ICT might be valuable prognostic tools for predicting OS and PFS and, thus, for the selection of patients with head and neck cancer who will benefit from CCRT.

## 1. Introduction

The first treatment method for every cancer is surgery. Unfortunately, head and neck squamous cell carcinomas (HNSCC) are not easy to resect and are associated with severe post-surgical complications and distortions of anatomical structures, which lead to worsening patients’ quality of life [1,2]. Furthermore, the prognosis of survival after surgery or induction chemotherapy in patients with stage III or IV of the disease is generally poor [3,4,5,6]. Therefore, other means of treatment are considered for these patients, such as concurrent chemoradiotherapy (CCRT) after induction chemotherapy (ICT) [7].

There are several reports in the literature suggesting that ICT may promote preservation of vital organs and support the quality of life. Few studies have shown associations between ICT and fewer distant metastases [8,9,10]. Published data demonstrate complete response (CR) after three cycles of ICT as a significant predictor for further disease control [7]. Some studies have shown that adding docetaxel to the scheme in patients with unresectable HNSCC improves survival [11,12]. Other studies have reported no benefits in terms of survival [13,14,15] compared with CCRT alone, except if ICT is given before treatment combined with delayed CCRT [16]. The published data we found are scattered, usually non-systematic and often even controversial. This proves that ICT is often used in clinical practice without definite scientific evidence.

Another important problem in clinical practice is ICT treatment response assessment. In most centers, the tumor’s response to ICT is assessed by using contrast-enhanced computed tomography (CECT) or magnetic resonance imaging (MRI) modalities. It is well known that response criteria of solid tumors (World Health Organisation (WHO) and Response evaluation criteria in solid tumors (RECIST)) based on a tumor size is not reliable due to difficulties of measurement caused by post-treatment necrosis, inflammation, etc. Therefore, researchers are still in search for more reliable imaging modality for ICT treatment response assessment and early detection of recurrence. 18-fluoro-2-deoxy-glucose positron emission tomography computed tomography (^18^F-FDG PET/CT treatment assessment is based on tumor glucose metabolism changes during the treatment course. Gavid et al.’s (2015) study demonstrated that pre-treatment or post-treatment ^18^F-FDG PET/CT uptake parameters such as standardized uptake value (SUV) and hypermetabolic tumor volume might be more advantageous and can predict response with higher sensitivity compared with morphological changes on CT [17]. Even more, several clinical studies have demonstrated that the same ^18^F-FDG PET/CT parameters may help to predict the tumor’s response to ICT and patients’ survival [5,10,17]. In addition, of course, ^18^F-FDG PET/CT is more effective in detecting head and neck cancer recurrence compared with CT or MRI [6].

Based on published data, we made an assumption that changes in ^18^F-FDG PET/CT parameters after ICT may be used to identify patients who will benefit from further treatment with CCRT. Therefore, the aim of this prospective study was to assess effectiveness of early prediction of CCRT outcome by means of interim ^18^F-FDG PET/CT in patients with unresectable HNSCC focusing on patient survival and clinical outcomes.

It is worth mentioning that there are only few published retrospective studies where both SUVmax and hypermetabolic tumor volume were included into treatment response evaluation [17,18].

## 2. Materials and Methods

### 2.1. Patients

From June 2013 to July 2016, a total of 49 patients with unresectable head and neck cancer were enrolled in a prospective study. Before staging, all patients underwent physical examination, panendoscopy and CECT. Staging was done according to the American Joint Committee on Cancer (8th edition). Patients with tumors limited to the oral cavity, oropharynx, hypoprarynx and larynx were selected for further investigation. The eligibility criteria were the following: histologically proven HNSCC at advanced stage III/IV and Eastern Cooperative Oncology Group performance status 0 or 1. The exclusion criteria were distant metastases at initial staging and previous history of head and neck cancer with previous chemotherapy and radiation. After ICT treatment, ^18^F-FDG PET/CT was not performed in 14 patients due to their health conditions; therefore, they were excluded from the study.

Prospective data compilations were approved by the Kaunas Regional Ethics Committee for Biomedical Research (No. BE-2-51; 5 November 2013). This study was registered at www.clinicaltrials.gov, number NCT02047201.

### 2.2. Therapeutic Principle

All patients received three cycles of induction chemotherapy: docetaxel 75 mg/m^2^, administered as an one-hour infusion on day 1, followed by cisplatin 75 mg/m^2^ administered as one-hour infusion on day 1, and 5-fluorouracil (5-FU) 750 mg/m^2^ per day, administered by continuous infusion from day 1 to day 5. Treatment was administered every three weeks (defined as one cycle). Reductions in the dose of docetaxel, cisplatin and 5-FU were performed dependent on patient’s tolerance and drug toxicity. All the patients underwent induction chemotherapy followed by CCRT. Within four weeks of the last ICT cycle, intensity modulated radiotherapy (IMRT) was initiated. The median target radiation dose for all patients who underwent IMRT was 61.6 Gy (range, 10.0–70.0 Gy). Intensity modulated radiotherapy fields were reduced for those who responded to ICT treatment. Reduction of radiotherapy fields should reduce amount and severity of treatment complications and increase the patients’ life quality with the same survival rate. The CCRT treatment consisted of cisplatin. The dose was modified according to the level of hematological toxicity of every patient, hepatic and renal function as well as the presence of infectious complications. Treatment toxicity during ICT and CCRT was evaluated weekly, as recommended by National Cancer Institute Common Toxicity Criteria (NCI CTCAE) v.4.0.

### 2.3. 18F-FDG PET/CT Examination

All the patients included in our study underwent two ^18^F-FDG PET/CT scans and CECT examinations: first for initial staging and second almost two weeks after the last cycle of ICT.

^18^F-FDG PET/CT examination consisted of a whole-body scan and a localized high-resolution head and neck scan. The patient’s preparation included fasting for more than 6 h. The serum glucose level on the day of the scan aimed to be <7 mmol/L. The injected activity of ^18^F-FDG was 4 MBq/kg of body weight. After injection, the patients remained in a quiet room for approximately 60 min.

A whole body ^18^F-FDG PET/CT scan was acquired (Discovery XCT, General Electric, Boston, MA, USA) (Discovery XCT, GE, USA) from the skull base to mid-thigh with patient’s hands above the head. The scan consisted of low-dose CT (120 kV, 100 mA, 3.75 mm section thickness) and PET acquisition time 3 min per bed position.

A localized head and neck ^18^F-FDG PET/CT scan was acquired with the patient positioned on a radiation therapy planning table with arms along the body. In addition, a post-ICT head and neck scan was performed while patients were immobilized in a treatment position with an individual thermoplastic mask, which extended to their shoulders. PET data were acquired for 5 min per bed position. Attenuation correction was performed using the CT data. The images were reconstructed in a 3-dimensional mode, using ordered subset expectation maximization algorithm (OSEM) with a 5 mm Gaussian filter on 128 × 128 and 256 × 256 matrices. A low-dose CT scan started from shoulders to the top of the head using 120 kV, 70 mA, and 3.75 mm.

### 2.4. Image Interpretation

Avid suspicious lesions in ^18^F-FDG were measured before and after ICT semi-quantitatively using SUVmax and hypermetabolic tumor volume [9]. The SUVmax region was drawn automatically by workstation tools. Visible border of hypermetabolic tumor volume was manually delineated using GE workstation instruments in three dimensions. Metabolic tumor response was evaluated corresponding to the SUVmax measurement and hypermetabolic tumor volume. The difference between two values of SUVmax and hypermetabolic tumor volume at two time points was calculated and expressed as percentage. According to literature the patients with SUVmax changes were classified as responders with decrease of ≥30% and non-responders when decrease was less than 30% [17]. Difference values were used to estimate correlations of concurrent chemoradiation treatment response and survival. SUVmax and hypermetabolic tumor volume variables were included into statistical analysis as prognostic factors for progression free survival (PFS) and overall survival (OS).

**Figure 1:**
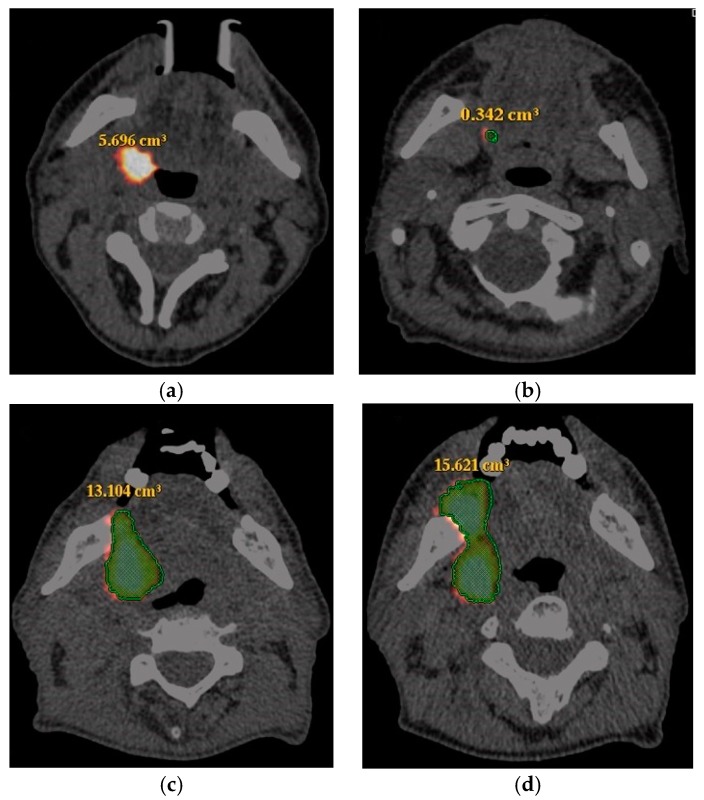
18-fluoro-2-deoxy-glucose positron emission tomography computed tomography performed at staging and after induction chemotherapy in patients with tonsils and tongue carcinoma. Hypermetabolic tumor alteration: (**a**,**b**) intense ^18^F-FDG uptake of the primary tumor and hypermetabolic tumor volume is reduced more than 90% (from 5.696 cm^3^ to 0.342 cm^3^)—these findings suggesting good response (progression-free survival (PFS) 26.9 and overall survival (OS) 26.9); (**c**,**d**) intense ^18^F-FDG uptake of the primary tumor and hypermetabolic tumor volume increased about 20% (from 13.104 cm^3^ to 15.621 cm^3^)—these findings suggesting progressive disease (PFS 6.1 and OS 7.4).

SUVmax changes after ICT treatment were estimated following PERCIST 1.0 criteria and response for all patients was defined as follows: complete metabolic response (CR), partial response (PR), stable disease (SD) and progressive disease (PD). The patients were divided into two groups: responders—when complete response or partial response was confirmed—and non-responders—when stable or progressive disease was diagnosed. The patients with hypermetabolic tumor volume changes were classified as responders with the volume decrease of ≥55% and non-responders when the decrease was less than 55% (Figure 1). Delta means were calculated by formula [(x(final)/x(primal)) × 100] − 100. Volume was express by cubic centimeters.

### 2.5. End Points

The primary end point of our study was progression-free survival (PFS), i.e., time from day 1 of the ICT first cycle to either disease progression or death. The secondary end point of our study was overall survival (OS), i.e., time from day 1 of the ICT first cycle until death from any reason or end of follow-up after 24 months. Study end points were established after complete treatment (ICT and CCRT).

### 2.6. Statistics

All statistical analyses were performed using Statistical Package for the Social Sciences (SPSS) version 22.0 for Windows. Normally distributed data are presented as mean and standard deviation (SD). Non-normally distributed data are expressed as median and range and were analyzed using non-parametric tests. The associations between ^18^F-FDG PET/CT parameters (SUVmax, hypermetabolic volume) were analyzed using the Fisher exact test. Differences among all study groups were evaluated by using the Mann-Whitney U test for independent samples. Survival estimates were evaluated by the Kaplan-Meier method and log-rank test. To assess the associations between survival and multiple clinicopathological and PET/CT variables, univariate and multivariate analysis was performed using the Cox proportional hazard model. The significance threshold was set at *p* < 0.05.

## 3. Results

### 3.1. Patient Characteristics

Patient characteristics at the time of diagnosis are listed in Table 1. Thirty-five patients with the mean age of 56.5 ± 8.6 years were included into the final statistical analysis. Twenty patients (51.4%) had oropharyngeal carcinoma, eighteen (46.2%) had hypopharyngeal and one had oral cavity carcinoma. Clinical stage T4 was the most common.

### 3.2. Response Evaluation

The median follow-up time was 18 months (range 5.4–34.6). Arranged by response to ICT, the distribution between classifications was significantly different between responders and non-responders. After induction chemotherapy, 11 (31.4%), 11 (31.4%), 9 (25.7%) and 4 (11.5%) patients demonstrated CR, PR, stable disease and progressive disease, respectively. Of the 11 patients who achieved CR, 2 patients died of disease progression. Of the 13 non-responders, 7 died of disease progression.

After induction chemotherapy, the median of SUVmax was 4.5 (range 2.2–12.0) for responders and 14.7 (range 4.0–21.6) for non-responders (*p* = 0.001). The median pre-treatment SUVmax was 16.4 (range 4.7–35.60) for responders and 19.1 (range 8.3–30.3) for non-responders (*p* = 0.878). The decrease median rate of SUVmax of HSNCC primary tumors was −76.86% (range −91.27 to −34.42) in the group of responders and −10.78% (range −19.75 to 142.2) in the group of non-responders.

**Figure 2:**
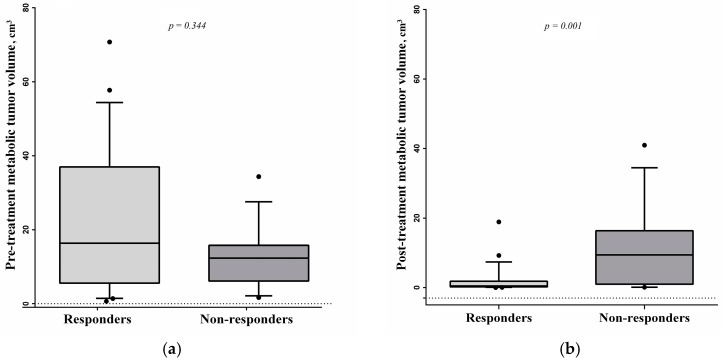
Relation between response to induction chemotherapy (ICT) with (**a**) pre-treatment and (**b**) post-treatment hypermetabolic tumor volume values in cubic centimeters.

The median pre-treatment and post-treatment hypermetabolic tumor volumes were 12.6 cm^3^ (range 0.7–70.8) and 0.71 cm^3^ (range 0.01–41). The association between pre-treatment and post-treatment hypermetabolic tumor volumes and the response to ICT is shown in Figure 2. The decrease rate of hypermetabolic tumor volume of HSNCC primary tumors was −99.6% to 754.4% (mean −91.3).

### 3.3. Survival Analysis

Considering that clinical outcome and ^18^F-FDG PET/CT variables may impact patient’s response assessment, univariate analysis of survival was performed. Based on univariate analysis: age, gender, tumor and node (TN) status, stage, differentiation and SUVmax groups showed no prognostic significance for overall survival. In contrast SUVmax and hypermetabolic tumor volume decrease changes were predictors for overall survival (*p* < 0.05). The results of univariate analysis are shown in Table 2.

A Cox multivariate regression analysis revealed that ICT treatment assessment in the primary tumors was an independent prognostic factor in OS (non-responders versus responders HR 7.489, 95% CI 1.633–34.44, *p* = 0.01). Hypermetabolic tumor volume and SUVmax values after ICT treatment were independent favorable predictive factors (respectively HR 1.004, 95% CI 1.001–1.008, *p* = 0.022 and HR 1.002, 95% CI 1.000–1.004, *p* = 0.02). SUVmax decrease <30% was independent prognostic factor of reduced overall survival (HR 1.059, 95% CI 1.00–2.59, *p* = 0.05) as well as hypermetabolic tumor volume decrease <55% HR 1.022, 95% CI 1.003–1.042, *p* = 0.026.

**Figure 3:**
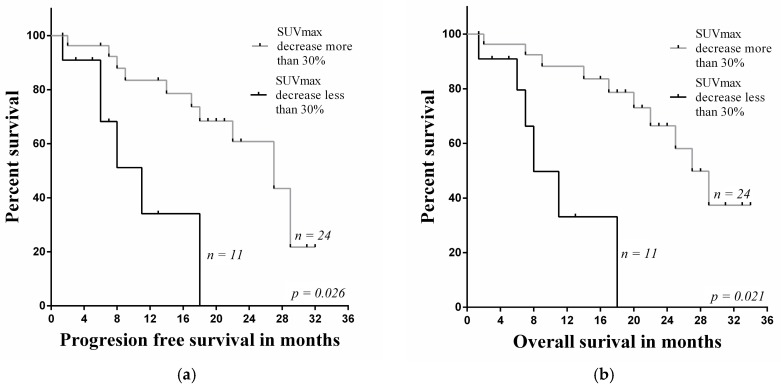
Comparison investigations of (**a**) PFS rate and (**b**) OS rate estimated by ^18^F-FDG PET/CT with SUVmax decrease in the primary lesion.

The decrease of SUVmax less than 30% in primary tumors evaluated by ^18^F-FDG PET/CT was a significant factor of poor prognosis in the group of non-responders for PFS and OS (*p* = 0.026, *p* = 0.021) (Figure 3). In non-responders group the median for PFS was 8.4 (range 6.1–28.4) and OS was 8.4 months (range 7.0–28.4).

Since there was no defined cut-off for the SUVmax value, the patients were divided into three groups according to pre-treatment SUVmax: lowest tertile (<10), medium (≥10 and <14.5) and high (≥14.5). Kaplan-Meier survival plots showed that the pre-treatment SUVmax middle group assessed by ^18^F-FDG PET/CT in primary tumors was significantly related to poor prognosis in PFS and OS (*p* = 0.001 and *p* = 0.006), respectively, when compared with the low and high SUVmax groups. After treatment, the patients were divided into two groups according to the follow-up SUVmax values: <5.3 and ≥5.3. Kaplan-Meier survival plots showed that post-treatment SUVmax values in the groups were not related to poor prognosis in PFS and OS (*p* = 0.396, *p* = 0.364).

**Figure 4:**
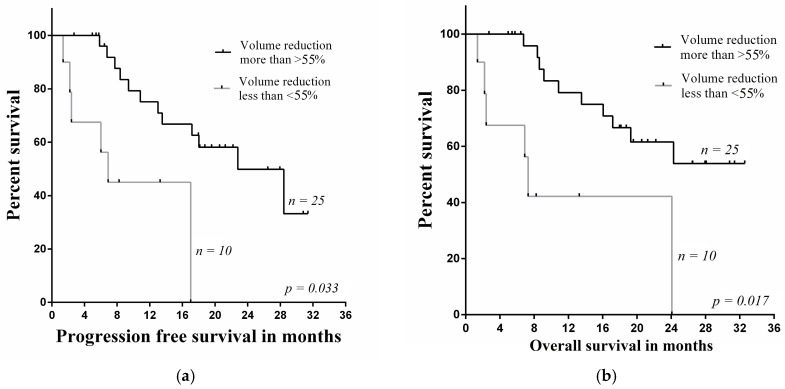
Comparison investigations of (**a**) PFS rate and (**b**) OS rate estimated by ^18^F-FDG PET/CT in the primary lesions using hypermetabolic tumor volume.

Kaplan-Meier survival plots showed that the hypermetabolic tumor volume of primary tumors in the group of non-responders assessed with ^18^F-FDG PET/CT decreased by less than 55%, and this insufficient decrease was significantly related to poor prognosis in PFS and OS (*p* = 0.033, *p* = 0.017) (Figure 4). In non-responders group the median for PFS was 7.7 (range 5.8–19.9) and OS was 7.9 months (range 5.8–24.4).

**Figure 5:**
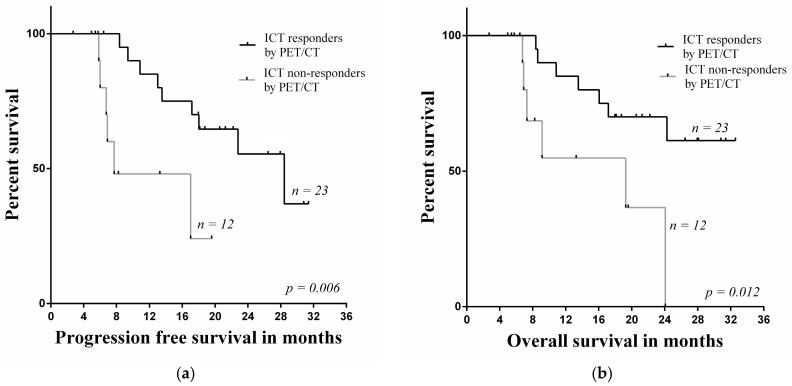
Comparison investigations of (**a**) PFS rate and (**b**) OS rate estimated by ^18^F-FDG PET/CT in the primary lesion.

Univariate analysis of survival plots showed that a decrease of SUVmax and hypermetabolic tumor volume of the primary tumor in the group of non-responders was significantly related to poor prognosis in PFS and OS (*p* = 0.006, *p* = 0.012). The median for PFS was 15.2 months (range 5.4–33.4) and the median for OS was 17.2 months (range 5.4–34.6) (Figure 5).

## 4. Discussion

Routine tumor staging, recurrence and prognosis are usually assessed on the basis of CT and MRI criteria. However, previous studies have suggested that it may be difficult to predict treatment outcomes, even among patients with the same stage of disease [5,19]. Furthermore, CT and MRI mostly detect anatomical changes, severely disturbed by necrosis and inflammation after treatment, which makes it almost impossible to detect a small amount of viable tumor among necrotic cells. Several studies have already reported that due to high ^18^F-FDG uptake in HNSCC it is possible to detect and differentiate metabolically active tumor cells among post-therapy changes [17,20,21]. Allegra et al. in their study had revealed a specificity, sensitivity and accuracy of PET/CT of 100%, 90% and 95.4% respectively, in patients with suspected metastatic disease [22]. Also, it has been noticed that changes of tumor metabolic activity as a reaction to ICT appear earlier than morphological changes [17,20]. Early treatment response assessment is very important in clinical practice, as it allows optimizing and individualizing the patient’s treatment, especially in cases where possible complications are very traumatic and worsening patient’s quality of life and overall survival. Thus, early prediction of treatment response before CCRT enables clinicians to choose different HNSCC therapy (surgery and radiotherapy/ CCRT), especially for patients with poor response to ICT.

The current study investigated the abilities of ^18^F-FDG PET/CT to prognosticate outcomes of following ICT as a predictor of the efficacy of CCRT in patients with stage III/IV HNSCC.

The data in the literature are scattered, and only a few studies have tried to identify response criteria after induction chemotherapy [7,23,24]; therefore, it is difficult to compare our results as different semi-quantitative prediction factors (SUVmax, SUVav, SUVlean, and SULmax) to evaluate prognosis are used in published data.

Our study was mainly focused on the efficacy of ^18^F-FDG PET/CT parameters (SUVmax and hypermetabolic tumor volume) and their ability to predict further outcome. Thestudy results support the idea that reduction in FDG uptake after therapy is linked to the outcome after CCRT. In order to demonstrate this, our study population was divided into three groups depending on their pre-treatment tumor SUVmax value. The patients with low metabolic activity tumors had respectively higher OS and PFS survival (*p* = 0.001, *p* = 0.006). Therefore, this makes it possible to predict that patients with a tumor of SUVmax <10 will respond to treatment and their overall quality of life will be much better compared with the other two groups (SUVmax > 10, but < 14.5 and SUVmax > 14.5). It seems that low metabolic activity tumors are more sensitive to induction chemotherapy and after all more sensitive to complete chemoradiotherapy treatment. We found it quite interesting that the middle group of patients (10 < SUVmax < 14.5) had significantly poorer prognosis in terms of PFS and OS compared with the other two groups (lowest SUVmax < 10 and highest SUVmax > 14.5). However, the study results might have been affected by relatively high morbidity compared with other groups and the small number of patients. A similar study was performed by Kawakita et al. (2013); they assigned patients into similar groups based on SUVmax intervals but did not detect any increased morbidity in the middle group [10]. We also aimed to define the cut-off value of SUVmax before and after ICT treatment between responders and non-responders. The patients in our study with lower SUVmax did not have significant differences in OS and PFS compared with patients with higher SUVmax. Thus, we were not able to establish a reliable SUVmax cut-off value and we do not recommend its use as a predictive factor. However, some trials using the median SUVmax of 5.5 as a cut-off value before treatment have shown a significant difference between groups of patients [19]. Allal et al. in their study (2004) tried to distinguish the mean SUVmax value after treatment. They found that patients with a median SUVmax <5.3 will respond to further treatment [25]. 

**Figure 6:**
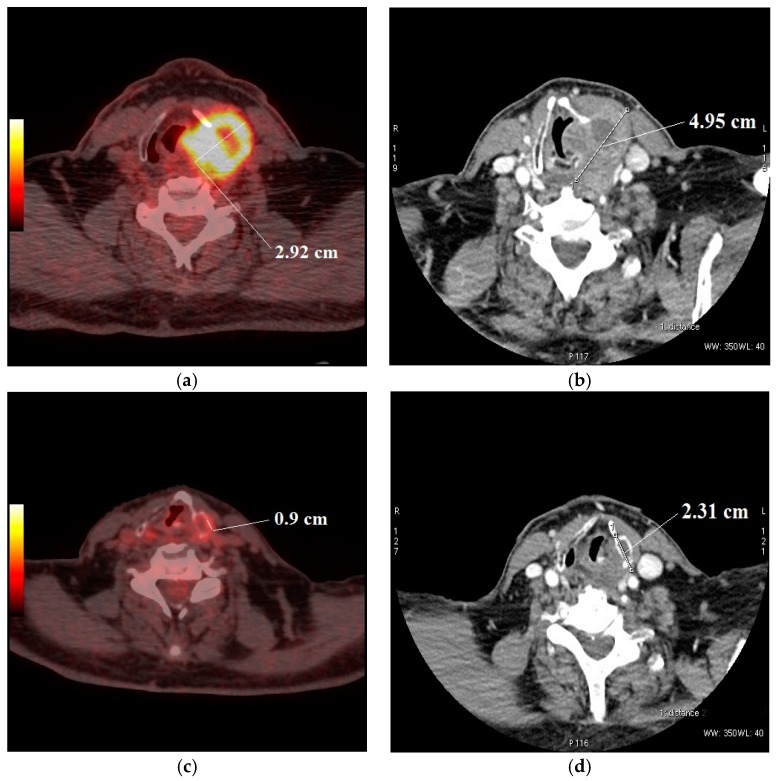
Contrast-enhanced CT and ^18^F-FDG PET/CT were performed at staging and after induction chemotherapy in patients with hypopharyngeal carcinoma. Tumor short axis alteration: (**a**,**c**) ^18^F-FDG metabolic uptake long axis before treatment 2.92 cm and after ICT—0.9 cm; (**b**,**d**) CT shows tumor short axis before treatment—4.95 cm and after treatment—2.31 cm.

The study results were also compared with those of Dalsaso et al. (2000) and Kikuchi et al. (2013), who also noted a significant relationship between the reduction of SUVmax and the tumor long axis on CT for determining therapeutic response [23,26]. The current investigation, although in a small number of patients, showed that about 70% to 73% of patients with identical reactions on both ^18^F-FDG PET/CT and CT demonstrated the same response to treatment. However, some studies have found relatively low correlations between CT or MRI and PET/CT [27,28,29], and a non-significant discrepancy in tumor-volume decrease on CT (*p* = 0.09) [30,31]. A clinical example is demonstrated in Figure 6. Nevertheless, most of the studies performing ^18^F-FDG PET/CT after CCRT suggest that PET/CT criteria might be a reliable indicator of the disease process.

Although tumor volume is not measured routinely by analysis of 18F-FDG PET/CT results, it might be a useful predictive factor. In this study, we demonstrated that hypermetabolic tumor volume measured after ICT can be used to select patients for further treatment and also predict the outcomes of CCRT. Furthermore, the PFS and OS rates were significantly lower in the group of non-responders (*p* = 0.033, *p* = 0.017, respectively). Our study results suggested that post-treatment hypermetabolic tumor volume and volume decrease might be significant predictive factors, especially in terms of predicting patient OS after CCRT. Gavid et al.’s (2015) study also aimed to measure tumor volume as a predictive factor. However, the researchers were unable to identify a significant volume threshold [17]. Tumor volumes could be an important factor in planning radiation therapy over tumor fields. It is worth mentioning that tumor volumes defined on PET scans were typically smaller than volumes defined on CT [29]. 

We found that the group of non-responders, which showed significantly poor PFS and OS when the primary tumor response was evaluated according to SUVmax, also showed a hypermetabolic tumor volume decrease. Thus, we cautiously suggest that patients with low or no metabolic response after ICT might have a poor chance of cure with CCRT. Yoon et al. (2011) have also noted that poor metabolic response was related to poor outcome following CCRT [7].

This study has some limitations. First, the outcome of HNSCC was not adjusted in relation to human papillomavirus (HPV), as the HPV tests in our institution are only performed in patients with oropharyngeal cancer. Second, the follow-up period was relatively short, but the study is still ongoing. The third limitation was related to the methodology, i.e., the second PET/CT was performed only 2 weeks after completion of ICT. This might have resulted in few false positive results due to treatment-related inflammation. Furthermore, a second biopsy was not performed in order to exclude possible inflammation. Only a few patients underwent the second biopsy at the very beginning of the study and it did not show any malignancy; therefore, it was decided not to repeat the biopsy after ICT treatment. Despite all these limitations, we derive significant results suggesting that hypermetabolic tumor volume and post-treatment SUVmax can be independent prognostic factors.

## 5. Conclusions

Our findings suggest that post-ICT treatment ^18^F-FDG PET/CT may predict the efficacy of CCRT in patients with HNSCC. We verified the prognostic relevance of pre-treatment hypermetabolic tumor volume obtained by ^18^F-FDG PET/CT. Hypermetabolic tumor volume decrease was closely related to prolonged PFS and OS.

## Figures and Tables

**Table 1:** Baseline patients and tumors characteristics.

Characteristics	Total, *n* (%) (*n* = 35)
Age (mean, years)	56.5 ± 8.6
Sex	
Male	33 (94.3)
Female	2 (5.7)
Stage of tumor	
T1	1 (2.9)
T2	7 (20)
T3	8 (22.9)
T4	19 (54.3)
Nodal stage	
N0–1	9 (25.7)
N2	25 (71.4)
N3	1 (2.9)
Overall stage	
III	6 (17.1)
IV	29 (82.9)
Histological grade	
G2	24 (68.6)
G3	11 (31.4)

**Table 2:** Univariate analysis of factors associated with overall survival.

Variables	Univariate Analysis		
	HR	95% CI	*p*
Age (<65 vs. ≥65)	1.402	0.306–6.414	0.663
Gender (female vs. male)	0.792	0.101–6.198	0.824
pT status (T1-2 vs T3-4)	0.676	0.148–3.089	0.613
N status (N0–1 vs. N2–3)	0.368	0.079–1.711	0.202
Stage (III vs. IV)	0.281	0.036–2.193	0.226
Differentiation (poor vs. moderate)	2.435	0.533–11.127	0.251
SUVmax (<30% vs. ≥30%)	0.228	0.073–0.715	0.011
SUVmax groups (<10 vs. >10 and <14.5 vs. ≥14.5)	0.580	0.176–1.914	0.371
SUVmax groups (<3.5 vs. ≥3.5)	0.033	0.001–11.414	0.253
Volume (<55% vs. ≥55%)	0.108	0.032–0.363	0.001
